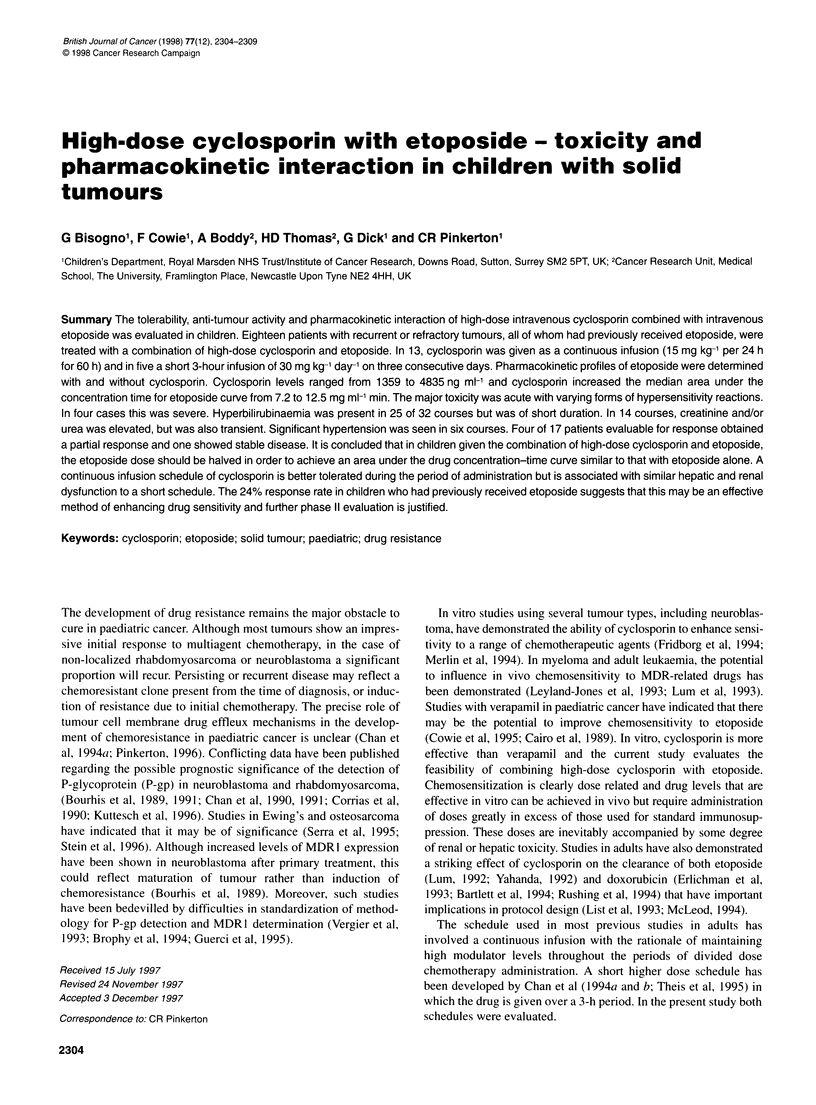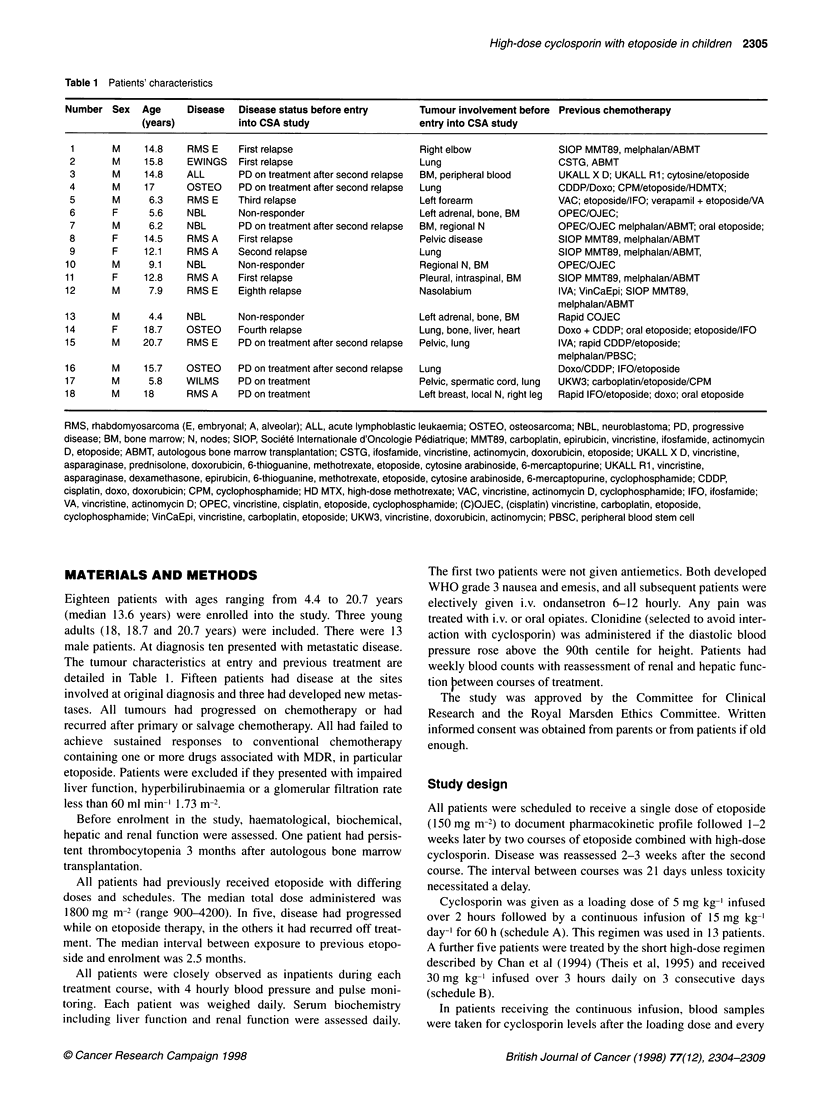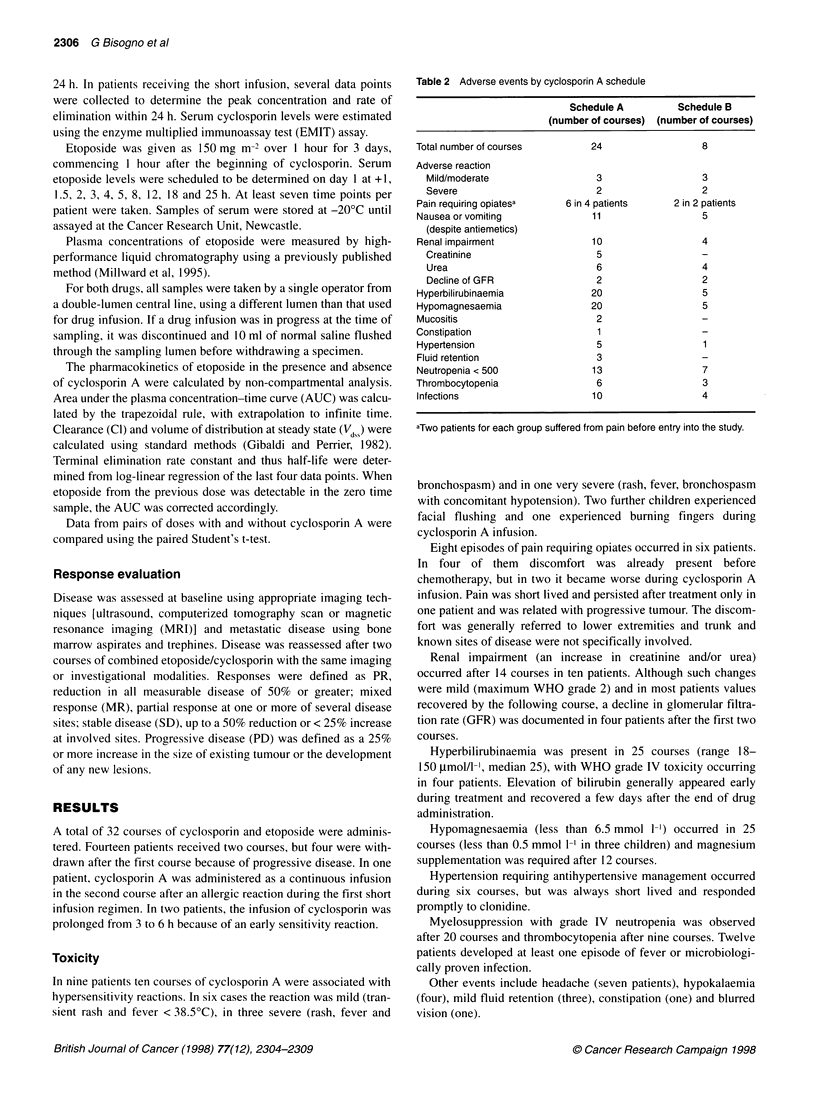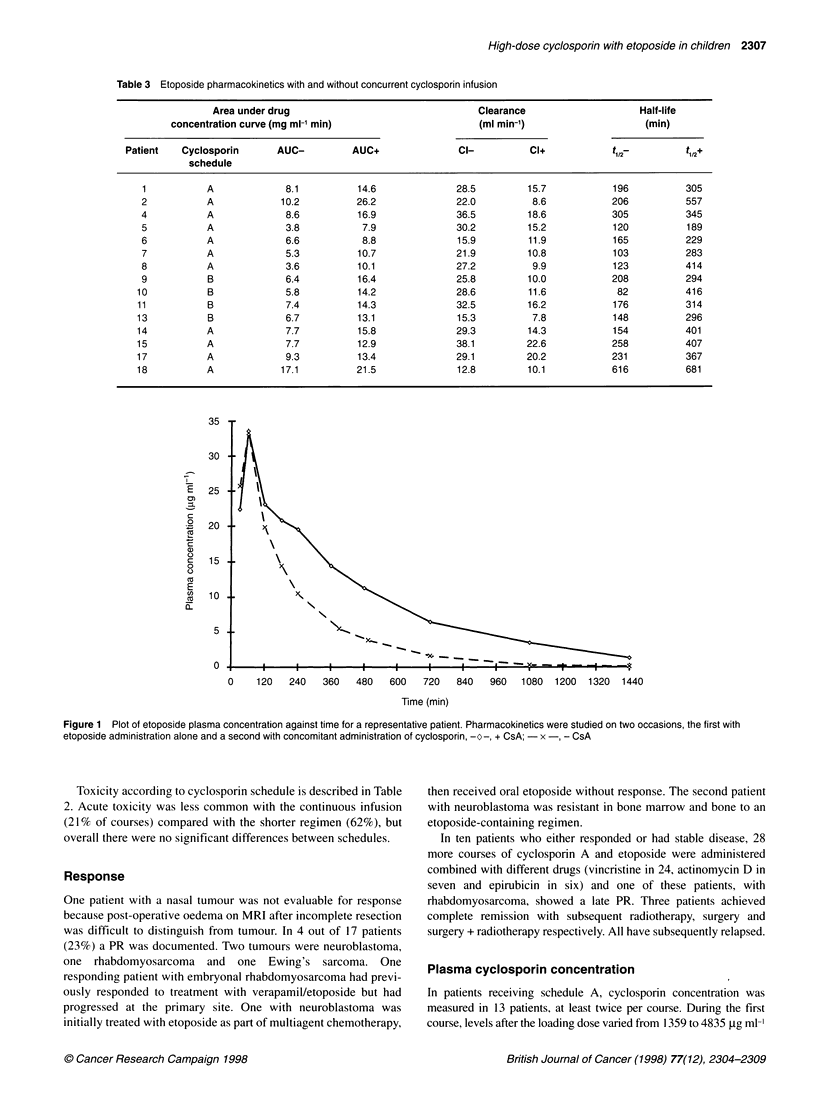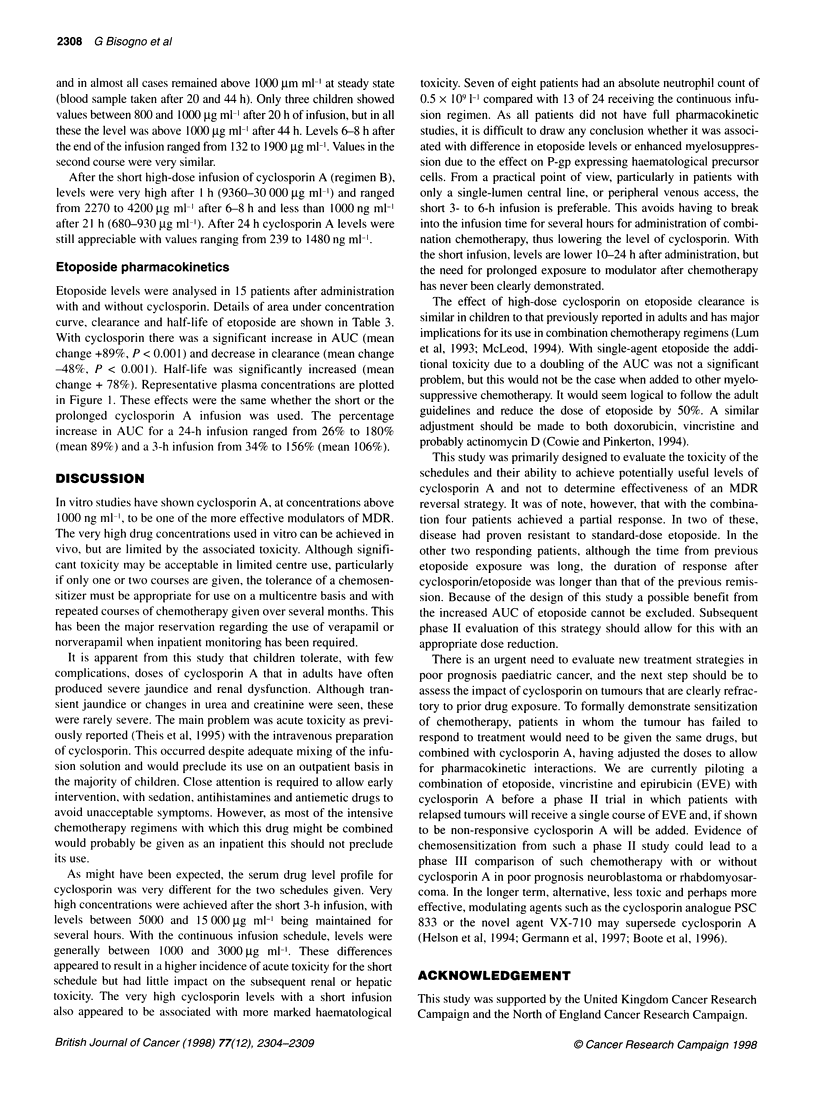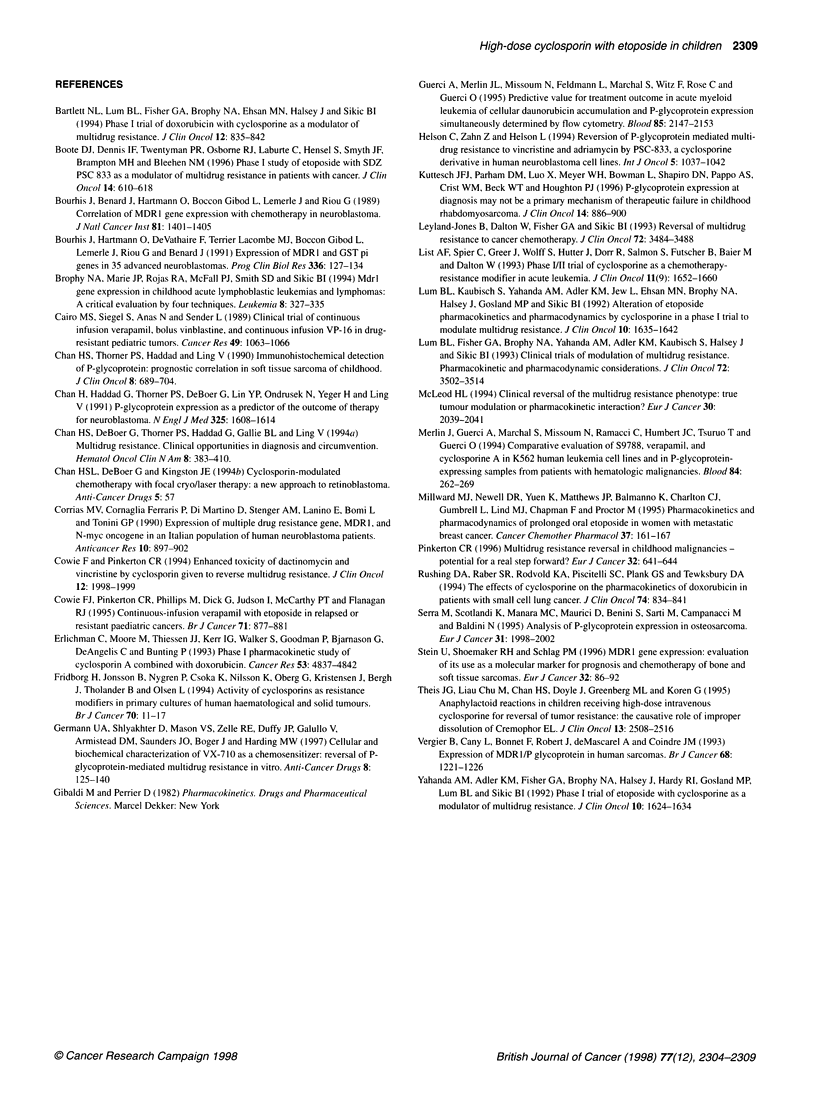# High-dose cyclosporin with etoposide--toxicity and pharmacokinetic interaction in children with solid tumours.

**DOI:** 10.1038/bjc.1998.383

**Published:** 1998-06

**Authors:** G. Bisogno, F. Cowie, A. Boddy, H. D. Thomas, G. Dick, C. R. Pinkerton

**Affiliations:** Children's Department, Royal Marsden NHS Trust/Institute of Cancer Research, Sutton, Surrey, UK.

## Abstract

The tolerability, anti-tumour activity and pharmacokinetic interaction of high-dose intravenous cyclosporin combined with intravenous etoposide was evaluated in children. Eighteen patients with recurrent or refractory tumours, all of whom had previously received etoposide, were treated with a combination of high-dose cyclosporin and etoposide. In 13, cyclosporin was given as a continuous infusion (15 mg kg(-1) per 24 h for 60 h) and in five a short 3-hour infusion of 30 mg kg(-1) day(-1) on three consecutive days. Pharmacokinetic profiles of etoposide were determined with and without cyclosporin. Cyclosporin levels ranged from 1359 to 4835 ng ml(-1) and cyclosporin increased the median area under the concentration time for etoposide curve from 7.2 to 12.5 mg ml(-1) min. The major toxicity was acute with varying forms of hypersensitivity reactions. In four cases this was severe. Hyperbilirubinaemia was present in 25 of 32 courses but was of short duration. In 14 courses, creatinine and/or urea was elevated, but was also transient. Significant hypertension was seen in six courses. Four of 17 patients evaluable for response obtained a partial response and one showed stable disease. It is concluded that in children given the combination of high-dose cyclosporin and etoposide, the etoposide dose should be halved in order to achieve an area under the drug concentration-time curve similar to that with etoposide alone. A continuous infusion schedule of cyclosporin is better tolerated during the period of administration but is associated with similar hepatic and renal dysfunction to a short schedule. The 24% response rate in children who had previously received etoposide suggests that this may be an effective method of enhancing drug sensitivity and further phase II evaluation is justified.


British Journal of Cancer (1998) 77(12), 2304–2309
© 1998 Cancer Research Campaign

### High-dose cyclosporin with etoposide – toxicity and pharmacokinetic interaction in children with solid tumours

**G Bisogno[1], F Cowie[1], A Boddy[2], HD Thomas[2], G Dick[1] and CR Pinkerton[1]**

[1]Children's Department, Royal Marsden NHS Trust/Institute of Cancer Research, Downs Road, Sutton, Surrey SM2 5PT, UK; [2]Cancer Research Unit, Medical School, The University, Framlington Place, Newcastle Upon Tyne NE2 4HH, UK

**Summary** The tolerability, anti-tumour activity and pharmacokinetic interaction of high-dose intravenous cyclosporin combined with intravenous etoposide was evaluated in children. Eighteen patients with recurrent or refractory tumours, all of whom had previously received etoposide, were treated with a combination of high-dose cyclosporin and etoposide. In 13, cyclosporin was given as a continuous infusion (15 mg $kg^{-1}$ per 24 h for 60 h) and in five a short 3-hour infusion of 30 mg $kg^{-1}$ $day^{-1}$ on three consecutive days. Pharmacokinetic profiles of etoposide were determined with and without cyclosporin. Cyclosporin levels ranged from 1359 to 4835 ng $ml^{-1}$ and cyclosporin increased the median area under the concentration time for etoposide curve from 7.2 to 12.5 mg $ml^{-1}$ min. The major toxicity was acute with varying forms of hypersensitivity reactions. In four cases this was severe. Hyperbilirubinaemia was present in 25 of 32 courses but was of short duration. In 14 courses, creatinine and/or urea was elevated, but was also transient. Significant hypertension was seen in six courses. Four of 17 patients evaluable for response obtained a partial response and one showed stable disease. It is concluded that in children given the combination of high-dose cyclosporin and etoposide, the etoposide dose should be halved in order to achieve an area under the drug concentration–time curve similar to that with etoposide alone. A continuous infusion schedule of cyclosporin is better tolerated during the period of administration but is associated with similar hepatic and renal dysfunction to a short schedule. The 24% response rate in children who had previously received etoposide suggests that this may be an effective method of enhancing drug sensitivity and further phase II evaluation is justified.

**Keywords:** cyclosporin; etoposide; solid tumour; paediatric; drug resistance

The development of drug resistance remains the major obstacle to cure in paediatric cancer. Although most tumours show an impressive initial response to multiagent chemotherapy, in the case of non-localized rhabdomyosarcoma or neuroblastoma a significant proportion will recur. Persisting or recurrent disease may reflect a chemoresistant clone present from the time of diagnosis, or induction of resistance due to initial chemotherapy. The precise role of tumour cell membrane drug effleux mechanisms in the development of chemoresistance in paediatric cancer is unclear (Chan et al, 1994*a*; Pinkerton, 1996). Conflicting data have been published regarding the possible prognostic significance of the detection of P-glycoprotein (P-gp) in neuroblastoma and rhabdomyosarcoma, (Bourhis et al, 1989, 1991; Chan et al, 1990, 1991; Corrias et al, 1990; Kuttesch et al, 1996). Studies in Ewing's and osteosarcoma have indicated that it may be of significance (Serra et al, 1995; Stein et al, 1996). Although increased levels of MDR1 expression have been shown in neuroblastoma after primary treatment, this could reflect maturation of tumour rather than induction of chemoresistance (Bourhis et al, 1989). Moreover, such studies have been bedevilled by difficulties in standardization of methodology for P-gp detection and MDR1 determination (Vergier et al, 1993; Brophy et al, 1994; Guerci et al, 1995).

*Received 15 July 1997*
*Revised 24 November 1997*
*Accepted 3 December 1997*

*Correspondence to:* CR Pinkerton

In vitro studies using several tumour types, including neuroblastoma, have demonstrated the ability of cyclosporin to enhance sensitivity to a range of chemotherapeutic agents (Fridborg et al, 1994; Merlin et al, 1994). In myeloma and adult leukaemia, the potential to influence in vivo chemosensitivity to MDR-related drugs has been demonstrated (Leyland-Jones et al, 1993; Lum et al, 1993). Studies with verapamil in paediatric cancer have indicated that there may be the potential to improve chemosensitivity to etoposide (Cowie et al, 1995; Cairo et al, 1989). In vitro, cyclosporin is more effective than verapamil and the current study evaluates the feasibility of combining high-dose cyclosporin with etoposide. Chemosensitization is clearly dose related and drug levels that are effective in vitro can be achieved in vivo but require administration of doses greatly in excess of those used for standard immunosuppression. These doses are inevitably accompanied by some degree of renal or hepatic toxicity. Studies in adults have also demonstrated a striking effect of cyclosporin on the clearance of both etoposide (Lum, 1992; Yahanda, 1992) and doxorubicin (Erlichman et al, 1993; Bartlett et al, 1994; Rushing et al, 1994) that have important implications in protocol design (List et al, 1993; McLeod, 1994).

The schedule used in most previous studies in adults has involved a continuous infusion with the rationale of maintaining high modulator levels throughout the periods of divided dose chemotherapy administration. A short higher dose schedule has been developed by Chan et al (1994*a* and *b*; Theis et al, 1995) in which the drug is given over a 3-h period. In the present study both schedules were evaluated.

**Table 1** Patients' characteristics

| Number | Sex | Age (years) | Disease | Disease status before entry into CSA study | Tumour involvement before entry into CSA study | Previous chemotherapy |
|---|---|---|---|---|---|---|
| 1 | M | 14.8 | RMS E | First relapse | Right elbow | SIOP MMT89, melphalan/ABMT |
| 2 | M | 15.8 | EWINGS | First relapse | Lung | CSTG, ABMT |
| 3 | M | 14.8 | ALL | PD on treatment after second relapse | BM, peripheral blood | UKALL X D; UKALL R1; cytosine/etoposide |
| 4 | M | 17 | OSTEO | PD on treatment after second relapse | Lung | CDDP/Doxo; CPM/etoposide/HDMTX; |
| 5 | M | 6.3 | RMS E | Third relapse | Left forearm | VAC; etoposide/IFO; verapamil + etoposide/VA |
| 6 | F | 5.6 | NBL | Non-responder | Left adrenal, bone, BM | OPEC/OJEC; |
| 7 | M | 6.2 | NBL | PD on treatment after second relapse | BM, regional N | OPEC/OJEC melphalan/ABMT; oral etoposide; |
| 8 | F | 14.5 | RMS A | First relapse | Pelvic disease | SIOP MMT89, melphalan/ABMT |
| 9 | F | 12.1 | RMS A | Second relapse | Lung | SIOP MMT89, melphalan/ABMT, |
| 10 | M | 9.1 | NBL | Non-responder | Regional N, BM | OPEC/OJEC |
| 11 | F | 12.8 | RMS A | First relapse | Pleural, intraspinal, BM | SIOP MMT89, melphalan/ABMT |
| 12 | M | 7.9 | RMS E | Eighth relapse | Nasolabium | IVA; VinCaEpi; SIOP MMT89, melphalan/ABMT |
| 13 | M | 4.4 | NBL | Non-responder | Left adrenal, bone, BM | Rapid COJEC |
| 14 | F | 18.7 | OSTEO | Fourth relapse | Lung, bone, liver, heart | Doxo + CDDP; oral etoposide; etoposide/IFO |
| 15 | M | 20.7 | RMS E | PD on treatment after second relapse | Pelvic, lung | IVA; rapid CDDP/etoposide; melphalan/PBSC; |
| 16 | M | 15.7 | OSTEO | PD on treatment after second relapse | Lung | Doxo/CDDP; IFO/etoposide |
| 17 | M | 5.8 | WILMS | PD on treatment | Pelvic, spermatic cord, lung | UKW3; carboplatin/etoposide/CPM |
| 18 | M | 18 | RMS A | PD on treatment | Left breast, local N, right leg | Rapid IFO/etoposide; doxo; oral etoposide |

RMS, rhabdomyosarcoma (E, embryonal; A, alveolar); ALL, acute lymphoblastic leukaemia; OSTEO, osteosarcoma; NBL, neuroblastoma; PD, progressive disease; BM, bone marrow; N, nodes; SIOP, Société Internationale d'Oncologie Pédiatrique; MMT89, carboplatin, epirubicin, vincristine, ifosfamide, actinomycin D, etoposide; ABMT, autologous bone marrow transplantation; CSTG, ifosfamide, vincristine, actinomycin, doxorubicin, etoposide; UKALL X D, vincristine, asparaginase, prednisolone, doxorubicin, 6-thioguanine, methotrexate, etoposide, cytosine arabinoside, 6-mercaptopurine; UKALL R1, vincristine, asparaginase, dexamethasone, epirubicin, 6-thioguanine, methotrexate, etoposide, cytosine arabinoside, 6-mercaptopurine, cyclophosphamide; CDDP, cisplatin, doxo, doxorubicin; CPM, cyclophosphamide; HD MTX, high-dose methotrexate; VAC, vincristine, actinomycin D, cyclophosphamide; IFO, ifosfamide; VA, vincristine, actinomycin D; OPEC, vincristine, cisplatin, etoposide, cyclophosphamide; (C)OJEC, (cisplatin) vincristine, carboplatin, etoposide, cyclophosphamide; VinCaEpi, vincristine, carboplatin, etoposide; UKW3, vincristine, doxorubicin, actinomycin; PBSC, peripheral blood stem cell

### MATERIALS AND METHODS

Eighteen patients with ages ranging from 4.4 to 20.7 years (median 13.6 years) were enrolled into the study. Three young adults (18, 18.7 and 20.7 years) were included. There were 13 male patients. At diagnosis ten presented with metastatic disease. The tumour characteristics at entry and previous treatment are detailed in Table 1. Fifteen patients had disease at the sites involved at original diagnosis and three had developed new metastases. All tumours had progressed on chemotherapy or had recurred after primary or salvage chemotherapy. All had failed to achieve sustained responses to conventional chemotherapy containing one or more drugs associated with MDR, in particular etoposide. Patients were excluded if they presented with impaired liver function, hyperbilirubinaemia or a glomerular filtration rate less than 60 ml $min^{-1}$ 1.73 $m^{-2}$.

Before enrolment in the study, haematological, biochemical, hepatic and renal function were assessed. One patient had persistent thrombocytopenia 3 months after autologous bone marrow transplantation.

All patients had previously received etoposide with differing doses and schedules. The median total dose administered was 1800 mg $m^{-2}$ (range 900–4200). In five, disease had progressed while on etoposide therapy, in the others it had recurred off treatment. The median interval between exposure to previous etoposide and enrolment was 2.5 months.

All patients were closely observed as inpatients during each treatment course, with 4 hourly blood pressure and pulse monitoring. Each patient was weighed daily. Serum biochemistry including liver function and renal function were assessed daily. The first two patients were not given antiemetics. Both developed WHO grade 3 nausea and emesis, and all subsequent patients were electively given i.v. ondansetron 6–12 hourly. Any pain was treated with i.v. or oral opiates. Clonidine (selected to avoid interaction with cyclosporin) was administered if the diastolic blood pressure rose above the 90th centile for height. Patients had weekly blood counts with reassessment of renal and hepatic function between courses of treatment.

The study was approved by the Committee for Clinical Research and the Royal Marsden Ethics Committee. Written informed consent was obtained from parents or from patients if old enough.

### Study design

All patients were scheduled to receive a single dose of etoposide (150 mg $m^{-2}$) to document pharmacokinetic profile followed 1–2 weeks later by two courses of etoposide combined with high-dose cyclosporin. Disease was reassessed 2–3 weeks after the second course. The interval between courses was 21 days unless toxicity necessitated a delay.

Cyclosporin was given as a loading dose of 5 mg $kg^{-1}$ infused over 2 hours followed by a continuous infusion of 15 mg $kg^{-1}$ $day^{-1}$ for 60 h (schedule A). This regimen was used in 13 patients. A further five patients were treated by the short high-dose regimen described by Chan et al (1994) (Theis et al, 1995) and received 30 mg $kg^{-1}$ infused over 3 hours daily on 3 consecutive days (schedule B).

In patients receiving the continuous infusion, blood samples were taken for cyclosporin levels after the loading dose and every

© Cancer Research Campaign 1998

24 h. In patients receiving the short infusion, several data points were collected to determine the peak concentration and rate of elimination within 24 h. Serum cyclosporin levels were estimated using the enzyme multiplied immunoassay test (EMIT) assay.

Etoposide was given as 150 mg $m^{-2}$ over 1 hour for 3 days, commencing 1 hour after the beginning of cyclosporin. Serum etoposide levels were scheduled to be determined on day 1 at +1, 1.5, 2, 3, 4, 5, 8, 12, 18 and 25 h. At least seven time points per patient were taken. Samples of serum were stored at –20°C until assayed at the Cancer Research Unit, Newcastle.

Plasma concentrations of etoposide were measured by high-performance liquid chromatography using a previously published method (Millward et al, 1995).

For both drugs, all samples were taken by a single operator from a double-lumen central line, using a different lumen than that used for drug infusion. If a drug infusion was in progress at the time of sampling, it was discontinued and 10 ml of normal saline flushed through the sampling lumen before withdrawing a specimen.

The pharmacokinetics of etoposide in the presence and absence of cyclosporin A were calculated by non-compartmental analysis. Area under the plasma concentration–time curve (AUC) was calculated by the trapezoidal rule, with extrapolation to infinite time. Clearance (Cl) and volume of distribution at steady state ($V_{dss}$) were calculated using standard methods (Gibaldi and Perrier, 1982). Terminal elimination rate constant and thus half-life were determined from log-linear regression of the last four data points. When etoposide from the previous dose was detectable in the zero time sample, the AUC was corrected accordingly.

Data from pairs of doses with and without cyclosporin A were compared using the paired Student's t-test.

### Response evaluation

Disease was assessed at baseline using appropriate imaging techniques [ultrasound, computerized tomography scan or magnetic resonance imaging (MRI)] and metastatic disease using bone marrow aspirates and trephines. Disease was reassessed after two courses of combined etoposide/cyclosporin with the same imaging or investigational modalities. Responses were defined as PR, reduction in all measurable disease of 50% or greater; mixed response (MR), partial response at one or more of several disease sites; stable disease (SD), up to a 50% reduction or < 25% increase at involved sites. Progressive disease (PD) was defined as a 25% or more increase in the size of existing tumour or the development of any new lesions.

### RESULTS

A total of 32 courses of cyclosporin and etoposide were administered. Fourteen patients received two courses, but four were withdrawn after the first course because of progressive disease. In one patient, cyclosporin A was administered as a continuous infusion in the second course after an allergic reaction during the first short infusion regimen. In two patients, the infusion of cyclosporin was prolonged from 3 to 6 h because of an early sensitivity reaction.

### Toxicity

In nine patients ten courses of cyclosporin A were associated with hypersensitivity reactions. In six cases the reaction was mild (transient rash and fever < 38.5°C), in three severe (rash, fever and bronchospasm) and in one very severe (rash, fever, bronchospasm with concomitant hypotension). Two further children experienced facial flushing and one experienced burning fingers during cyclosporin A infusion.

**Table 2** Adverse events by cyclosporin A schedule

| | Schedule A (number of courses) | Schedule B (number of courses) |
|---|---|---|
| Total number of courses | 24 | 8 |
| Adverse reaction | | |
| Mild/moderate | 3 | 3 |
| Severe | 2 | 2 |
| Pain requiring opiates[a] | 6 in 4 patients | 2 in 2 patients |
| Nausea or vomiting (despite antiemetics) | 11 | 5 |
| Renal impairment | 10 | 4 |
| Creatinine | 5 | – |
| Urea | 6 | 4 |
| Decline of GFR | 2 | 2 |
| Hyperbilirubinaemia | 20 | 5 |
| Hypomagnesaemia | 20 | 5 |
| Mucositis | 2 | – |
| Constipation | 1 | – |
| Hypertension | 5 | 1 |
| Fluid retention | 3 | – |
| Neutropenia < 500 | 13 | 7 |
| Thrombocytopenia | 6 | 3 |
| Infections | 10 | 4 |

[a]Two patients for each group suffered from pain before entry into the study.

Eight episodes of pain requiring opiates occurred in six patients. In four of them discomfort was already present before chemotherapy, but in two it became worse during cyclosporin A infusion. Pain was short lived and persisted after treatment only in one patient and was related with progressive tumour. The discomfort was generally referred to lower extremities and trunk and known sites of disease were not specifically involved.

Renal impairment (an increase in creatinine and/or urea) occurred after 14 courses in ten patients. Although such changes were mild (maximum WHO grade 2) and in most patients values recovered by the following course, a decline in glomerular filtration rate (GFR) was documented in four patients after the first two courses.

Hyperbilirubinaemia was present in 25 courses (range 18–150 μmol/$l^{-1}$, median 25), with WHO grade IV toxicity occurring in four patients. Elevation of bilirubin generally appeared early during treatment and recovered a few days after the end of drug administration.

Hypomagnesaemia (less than 6.5 mmol $l^{-1}$) occurred in 25 courses (less than 0.5 mmol $l^{-1}$ in three children) and magnesium supplementation was required after 12 courses.

Hypertension requiring antihypertensive management occurred during six courses, but was always short lived and responded promptly to clonidine.

Myelosuppression with grade IV neutropenia was observed after 20 courses and thrombocytopenia after nine courses. Twelve patients developed at least one episode of fever or microbiologically proven infection.

Other events include headache (seven patients), hypokalaemia (four), mild fluid retention (three), constipation (one) and blurred vision (one).

© Cancer Research Campaign 1998

**Table 3** Etoposide pharmacokinetics with and without concurrent cyclosporin infusion

| Area under drug concentration curve (mg ml$^{-1}$ min) | | | | Clearance (ml min$^{-1}$) | | Half-life (min) | |
|---|---|---|---|---|---|---|---|
| Patient | Cyclosporin schedule | AUC− | AUC+ | Cl− | Cl+ | $t_{1/2}$− | $t_{1/2}$+ |
| 1 | A | 8.1 | 14.6 | 28.5 | 15.7 | 196 | 305 |
| 2 | A | 10.2 | 26.2 | 22.0 | 8.6 | 206 | 557 |
| 4 | A | 8.6 | 16.9 | 36.5 | 18.6 | 305 | 345 |
| 5 | A | 3.8 | 7.9 | 30.2 | 15.2 | 120 | 189 |
| 6 | A | 6.6 | 8.8 | 15.9 | 11.9 | 165 | 229 |
| 7 | A | 5.3 | 10.7 | 21.9 | 10.8 | 103 | 283 |
| 8 | A | 3.6 | 10.1 | 27.2 | 9.9 | 123 | 414 |
| 9 | B | 6.4 | 16.4 | 25.8 | 10.0 | 208 | 294 |
| 10 | B | 5.8 | 14.2 | 28.6 | 11.6 | 82 | 416 |
| 11 | B | 7.4 | 14.3 | 32.5 | 16.2 | 176 | 314 |
| 13 | B | 6.7 | 13.1 | 15.3 | 7.8 | 148 | 296 |
| 14 | A | 7.7 | 15.8 | 29.3 | 14.3 | 154 | 401 |
| 15 | A | 7.7 | 12.9 | 38.1 | 22.6 | 258 | 407 |
| 17 | A | 9.3 | 13.4 | 29.1 | 20.2 | 231 | 367 |
| 18 | A | 17.1 | 21.5 | 12.8 | 10.1 | 616 | 681 |

**Figure 1** Plot of etoposide plasma concentration against time for a representative patient. Pharmacokinetics were studied on two occasions, the first with etoposide administration alone and a second with concomitant administration of cyclosporin, –◇–, + CsA; — × —, – CsA

Toxicity according to cyclosporin schedule is described in Table 2. Acute toxicity was less common with the continuous infusion (21% of courses) compared with the shorter regimen (62%), but overall there were no significant differences between schedules.

### Response

One patient with a nasal tumour was not evaluable for response because post-operative oedema on MRI after incomplete resection was difficult to distinguish from tumour. In 4 out of 17 patients (23%) a PR was documented. Two tumours were neuroblastoma, one rhabdomyosarcoma and one Ewing's sarcoma. One responding patient with embryonal rhabdomyosarcoma had previously responded to treatment with verapamil/etoposide but had progressed at the primary site. One with neuroblastoma was initially treated with etoposide as part of multiagent chemotherapy, then received oral etoposide without response. The second patient with neuroblastoma was resistant in bone marrow and bone to an etoposide-containing regimen.

In ten patients who either responded or had stable disease, 28 more courses of cyclosporin A and etoposide were administered combined with different drugs (vincristine in 24, actinomycin D in seven and epirubicin in six) and one of these patients, with rhabdomyosarcoma, showed a late PR. Three patients achieved complete remission with subsequent radiotherapy, surgery and surgery + radiotherapy respectively. All have subsequently relapsed.

### Plasma cyclosporin concentration

In patients receiving schedule A, cyclosporin concentration was measured in 13 patients, at least twice per course. During the first course, levels after the loading dose varied from 1359 to 4835 µg ml$^{-1}$

© Cancer Research Campaign 1998

and in almost all cases remained above 1000 µm ml$^{-1}$ at steady state (blood sample taken after 20 and 44 h). Only three children showed values between 800 and 1000 µg ml$^{-1}$ after 20 h of infusion, but in all these the level was above 1000 µg ml$^{-1}$ after 44 h. Levels 6–8 h after the end of the infusion ranged from 132 to 1900 µg ml$^{-1}$. Values in the second course were very similar.

After the short high-dose infusion of cyclosporin A (regimen B), levels were very high after 1 h (9360–30 000 µg ml$^{-1}$) and ranged from 2270 to 4200 µg ml$^{-1}$ after 6–8 h and less than 1000 ng ml$^{-1}$ after 21 h (680–930 µg ml$^{-1}$). After 24 h cyclosporin A levels were still appreciable with values ranging from 239 to 1480 ng ml$^{-1}$.

### Etoposide pharmacokinetics

Etoposide levels were analysed in 15 patients after administration with and without cyclosporin. Details of area under concentration curve, clearance and half-life of etoposide are shown in Table 3. With cyclosporin there was a significant increase in AUC (mean change +89%, $P < 0.001$) and decrease in clearance (mean change –48%, $P < 0.001$). Half-life was significantly increased (mean change + 78%). Representative plasma concentrations are plotted in Figure 1. These effects were the same whether the short or the prolonged cyclosporin A infusion was used. The percentage increase in AUC for a 24-h infusion ranged from 26% to 180% (mean 89%) and a 3-h infusion from 34% to 156% (mean 106%).

### DISCUSSION

In vitro studies have shown cyclosporin A, at concentrations above 1000 ng ml$^{-1}$, to be one of the more effective modulators of MDR. The very high drug concentrations used in vitro can be achieved in vivo, but are limited by the associated toxicity. Although significant toxicity may be acceptable in limited centre use, particularly if only one or two courses are given, the tolerance of a chemosensitizer must be appropriate for use on a multicentre basis and with repeated courses of chemotherapy given over several months. This has been the major reservation regarding the use of verapamil or norverapamil when inpatient monitoring has been required.

It is apparent from this study that children tolerate, with few complications, doses of cyclosporin A that in adults have often produced severe jaundice and renal dysfunction. Although transient jaundice or changes in urea and creatinine were seen, these were rarely severe. The main problem was acute toxicity as previously reported (Theis et al, 1995) with the intravenous preparation of cyclosporin. This occurred despite adequate mixing of the infusion solution and would preclude its use on an outpatient basis in the majority of children. Close attention is required to allow early intervention, with sedation, antihistamines and antiemetic drugs to avoid unacceptable symptoms. However, as most of the intensive chemotherapy regimens with which this drug might be combined would probably be given as an inpatient this should not preclude its use.

As might have been expected, the serum drug level profile for cyclosporin was very different for the two schedules given. Very high concentrations were achieved after the short 3-h infusion, with levels between 5000 and 15 000 µg ml$^{-1}$ being maintained for several hours. With the continuous infusion schedule, levels were generally between 1000 and 3000 µg ml$^{-1}$. These differences appeared to result in a higher incidence of acute toxicity for the short schedule but had little impact on the subsequent renal or hepatic toxicity. The very high cyclosporin levels with a short infusion also appeared to be associated with more marked haematological toxicity. Seven of eight patients had an absolute neutrophil count of $0.5 \times 10^9$ l$^{-1}$ compared with 13 of 24 receiving the continuous infusion regimen. As all patients did not have full pharmacokinetic studies, it is difficult to draw any conclusion whether it was associated with difference in etoposide levels or enhanced myelosuppression due to the effect on P-gp expressing haematological precursor cells. From a practical point of view, particularly in patients with only a single-lumen central line, or peripheral venous access, the short 3- to 6-h infusion is preferable. This avoids having to break into the infusion time for several hours for administration of combination chemotherapy, thus lowering the level of cyclosporin. With the short infusion, levels are lower 10–24 h after administration, but the need for prolonged exposure to modulator after chemotherapy has never been clearly demonstrated.

The effect of high-dose cyclosporin on etoposide clearance is similar in children to that previously reported in adults and has major implications for its use in combination chemotherapy regimens (Lum et al, 1993; McLeod, 1994). With single-agent etoposide the additional toxicity due to a doubling of the AUC was not a significant problem, but this would not be the case when added to other myelosuppressive chemotherapy. It would seem logical to follow the adult guidelines and reduce the dose of etoposide by 50%. A similar adjustment should be made to both doxorubicin, vincristine and probably actinomycin D (Cowie and Pinkerton, 1994).

This study was primarily designed to evaluate the toxicity of the schedules and their ability to achieve potentially useful levels of cyclosporin A and not to determine effectiveness of an MDR reversal strategy. It was of note, however, that with the combination four patients achieved a partial response. In two of these, disease had proven resistant to standard-dose etoposide. In the other two responding patients, although the time from previous etoposide exposure was long, the duration of response after cyclosporin/etoposide was longer than that of the previous remission. Because of the design of this study a possible benefit from the increased AUC of etoposide cannot be excluded. Subsequent phase II evaluation of this strategy should allow for this with an appropriate dose reduction.

There is an urgent need to evaluate new treatment strategies in poor prognosis paediatric cancer, and the next step should be to assess the impact of cyclosporin on tumours that are clearly refractory to prior drug exposure. To formally demonstrate sensitization of chemotherapy, patients in whom the tumour has failed to respond to treatment would need to be given the same drugs, but combined with cyclosporin A, having adjusted the doses to allow for pharmacokinetic interactions. We are currently piloting a combination of etoposide, vincristine and epirubicin (EVE) with cyclosporin A before a phase II trial in which patients with relapsed tumours will receive a single course of EVE and, if shown to be non-responsive cyclosporin A will be added. Evidence of chemosensitization from such a phase II study could lead to a phase III comparison of such chemotherapy with or without cyclosporin A in poor prognosis neuroblastoma or rhabdomyosarcoma. In the longer term, alternative, less toxic and perhaps more effective, modulating agents such as the cyclosporin analogue PSC 833 or the novel agent VX-710 may supersede cyclosporin A (Helson et al, 1994; Germann et al, 1997; Boote et al, 1996).

### ACKNOWLEDGEMENT

This study was supported by the United Kingdom Cancer Research Campaign and the North of England Cancer Research Campaign.

*© Cancer Research Campaign 1998*

### REFERENCES

Bartlett NL, Lum BL, Fisher GA, Brophy NA, Ehsan MN, Halsey J and Sikic BI (1994) Phase I trial of doxorubicin with cyclosporine as a modulator of multidrug resistance. *J Clin Oncol* **12**: 835–842

Boote DJ, Dennis IF, Twentyman PR, Osborne RJ, Laburte C, Hensel S, Smyth JF, Brampton MH and Bleehen NM (1996) Phase I study of etoposide with SDZ PSC 833 as a modulator of multidrug resistance in patients with cancer. *J Clin Oncol* **14**: 610–618

Bourhis J, Benard J, Hartmann O, Boccon Gibod L, Lemerle J and Riou G (1989) Correlation of MDR1 gene expression with chemotherapy in neuroblastoma. *J Natl Cancer Inst* **81**: 1401–1405

Bourhis J, Hartmann O, DeVathaire F, Terrier Lacombe MJ, Boccon Gibod L, Lemerle J, Riou G and Benard J (1991) Expression of MDR1 and GST pi genes in 35 advanced neuroblastomas. *Prog Clin Biol Res* **336**: 127–134

Brophy NA, Marie JP, Rojas RA, McFall PJ, Smith SD and Sikic BI (1994) Mdr1 gene expression in childhood acute lymphoblastic leukemias and lymphomas: A critical evaluation by four techniques. *Leukemia* **8**: 327–335

Cairo MS, Siegel S, Anas N and Sender L (1989) Clinical trial of continuous infusion verapamil, bolus vinblastine, and continuous infusion VP-16 in drug-resistant pediatric tumors. *Cancer Res* **49**: 1063–1066

Chan HS, Thorner PS, Haddad and Ling V (1990) Immunohistochemical detection of P-glycoprotein: prognostic correlation in soft tissue sarcoma of childhood. *J Clin Oncol* **8**: 689–704.

Chan H, Haddad G, Thorner PS, DeBoer G, Lin YP, Ondrusek N, Yeger H and Ling V (1991) P-glycoprotein expression as a predictor of the outcome of therapy for neuroblastoma. *N Engl J Med* **325**: 1608–1614

Chan HS, DeBoer G, Thorner PS, Haddad G, Gallie BL and Ling V (1994*a*) Multidrug resistance. Clinical opportunities in diagnosis and circumvention. *Hematol Oncol Clin N Am* **8**: 383–410.

Chan HSL, DeBoer G and Kingston JE (1994*b*) Cyclosporin-modulated chemotherapy with focal cryo/laser therapy: a new approach to retinoblastoma. *Anti-Cancer Drugs* **5**: 57

Corrias MV, Cornaglia Ferraris P, Di Martino D, Stenger AM, Lanino E, Bomi L and Tonini GP (1990) Expression of multiple drug resistance gene, MDR1, and N-myc oncogene in an Italian population of human neuroblastoma patients. *Anticancer Res* **10**: 897–902

Cowie F and Pinkerton CR (1994) Enhanced toxicity of dactinomycin and vincristine by cyclosporin given to reverse multidrug resistance. *J Clin Oncol* **12**: 1998–1999

Cowie FJ, Pinkerton CR, Phillips M, Dick G, Judson I, McCarthy PT and Flanagan RJ (1995) Continuous-infusion verapamil with etoposide in relapsed or resistant paediatric cancers. *Br J Cancer* **71**: 877–881

Erlichman C, Moore M, Thiessen JJ, Kerr IG, Walker S, Goodman P, Bjarnason G, DeAngelis C and Bunting P (1993) Phase I pharmacokinetic study of cyclosporin A combined with doxorubicin. *Cancer Res* **53**: 4837–4842

Fridborg H, Jonsson B, Nygren P, Csoka K, Nilsson K, Oberg G, Kristensen J, Bergh J, Tholander B and Olsen L (1994) Activity of cyclosporins as resistance modifiers in primary cultures of human haematological and solid tumours. *Br J Cancer* **70**: 11–17

Germann UA, Shlyakhter D, Mason VS, Zelle RE, Duffy JP, Galullo V, Armistead DM, Saunders JO, Boger J and Harding MW (1997) Cellular and biochemical characterization of VX-710 as a chemosensitizer: reversal of P-glycoprotein-mediated multidrug resistance in vitro. *Anti-Cancer Drugs* **8**: 125–140

Gibaldi M and Perrier D (1982) *Pharmacokinetics. Drugs and Pharmaceutical Sciences*. Marcel Dekker: New York

Guerci A, Merlin JL, Missoum N, Feldmann L, Marchal S, Witz F, Rose C and Guerci O (1995) Predictive value for treatment outcome in acute myeloid leukemia of cellular daunorubicin accumulation and P-glycoprotein expression simultaneously determined by flow cytometry. *Blood* **85**: 2147–2153

Helson C, Zahn Z and Helson L (1994) Reversion of P-glycoprotein mediated multi-drug resistance to vincristine and adriamycin by PSC-833, a cyclosporine derivative in human neuroblastoma cell lines. *Int J Oncol* **5**: 1037–1042

Kuttesch JFJ, Parham DM, Luo X, Meyer WH, Bowman L, Shapiro DN, Pappo AS, Crist WM, Beck WT and Houghton PJ (1996) P-glycoprotein expression at diagnosis may not be a primary mechanism of therapeutic failure in childhood rhabdomyosarcoma. *J Clin Oncol* **14**: 886–900

Leyland-Jones B, Dalton W, Fisher GA and Sikic BI (1993) Reversal of multidrug resistance to cancer chemotherapy. *J Clin Oncol* **72**: 3484–3488

List AF, Spier C, Greer J, Wolff S, Hutter J, Dorr R, Salmon S, Futscher B, Baier M and Dalton W (1993) Phase I/II trial of cyclosporine as a chemotherapy-resistance modifier in acute leukemia. *J Clin Oncol* **11**(9): 1652–1660

Lum BL, Kaubisch S, Yahanda AM, Adler KM, Jew L, Ehsan MN, Brophy NA, Halsey J, Gosland MP and Sikic BI (1992) Alteration of etoposide pharmacokinetics and pharmacodynamics by cyclosporine in a phase I trial to modulate multidrug resistance. *J Clin Oncol* **10**: 1635–1642

Lum BL, Fisher GA, Brophy NA, Yahanda AM, Adler KM, Kaubisch S, Halsey J and Sikic BI (1993) Clinical trials of modulation of multidrug resistance. Pharmacokinetic and pharmacodynamic considerations. *J Clin Oncol* **72**: 3502–3514

McLeod HL (1994) Clinical reversal of the multidrug resistance phenotype: true tumour modulation or pharmacokinetic interaction? *Eur J Cancer* **30**: 2039–2041

Merlin J, Guerci A, Marchal S, Missoum N, Ramacci C, Humbert JC, Tsuruo T and Guerci O (1994) Comparative evaluation of S9788, verapamil, and cyclosporine A in K562 human leukemia cell lines and in P-glycoprotein-expressing samples from patients with hematologic malignancies. *Blood* **84**: 262–269

Millward MJ, Newell DR, Yuen K, Matthews JP, Balmanno K, Charlton CJ, Gumbrell L, Lind MJ, Chapman F and Proctor M (1995) Pharmacokinetics and pharmacodynamics of prolonged oral etoposide in women with metastatic breast cancer. *Cancer Chemother Pharmacol* **37**: 161–167

Pinkerton CR (1996) Multidrug resistance reversal in childhood malignancies – potential for a real step forward? *Eur J Cancer* **32**: 641–644

Rushing DA, Raber SR, Rodvold KA, Piscitelli SC, Plank GS and Tewksbury DA (1994) The effects of cyclosporine on the pharmacokinetics of doxorubicin in patients with small cell lung cancer. *J Clin Oncol* **74**: 834–841

Serra M, Scotlandi K, Manara MC, Maurici D, Benini S, Sarti M, Campanacci M and Baldini N (1995) Analysis of P-glycoprotein expression in osteosarcoma. *Eur J Cancer* **31**: 1998–2002

Stein U, Shoemaker RH and Schlag PM (1996) MDR1 gene expression: evaluation of its use as a molecular marker for prognosis and chemotherapy of bone and soft tissue sarcomas. *Eur J Cancer* **32**: 86–92

Theis JG, Liau Chu M, Chan HS, Doyle J, Greenberg ML and Koren G (1995) Anaphylactoid reactions in children receiving high-dose intravenous cyclosporine for reversal of tumor resistance: the causative role of improper dissolution of Cremophor EL. *J Clin Oncol* **13**: 2508–2516

Vergier B, Cany L, Bonnet F, Robert J, deMascarel A and Coindre JM (1993) Expression of MDR1/P glycoprotein in human sarcomas. *Br J Cancer* **68**: 1221–1226

Yahanda AM, Adler KM, Fisher GA, Brophy NA, Halsey J, Hardy RI, Gosland MP, Lum BL and Sikic BI (1992) Phase I trial of etoposide with cyclosporine as a modulator of multidrug resistance. *J Clin Oncol* **10**: 1624–1634

© Cancer Research Campaign 1998